# Answers to Questions Posed During Daily Patient Care Are More Likely to Be Answered by UpToDate Than PubMed

**DOI:** 10.2196/jmir.1012

**Published:** 2008-10-03

**Authors:** Arjen Hoogendam, Anton FH Stalenhoef, Pieter F de Vries Robbé, A John PM Overbeke

**Affiliations:** ^2^Department of Medical InformaticsRadboud University NijmegenNijmegenThe Netherlands; ^1^Department of MedicineDivision of General Internal MedicineRadboud University Nijmegen Medical CentreNijmegenThe Netherlands

**Keywords:** PubMed, information storage and retrieval, evidence-based medicine, medical informatics, information services, Internet, hospitalists

## Abstract

**Background:**

UpToDate and PubMed are popular sources for medical information. Data regarding the efficiency of PubMed and UpToDate in daily medical care are lacking.

**Objective:**

The purpose of this observational study was to describe the percentage of answers retrieved by these information sources, comparing search results with regard to different medical topics and the time spent searching for an answer.

**Methods:**

A total of 40 residents and 30 internists in internal medicine working in an academic medical center searched PubMed and UpToDate using an observation portal during daily medical care. The information source used for searching and the time needed to find an answer to the question were recorded by the portal. Information was provided by searchers regarding the topic of the question, the situation that triggered the question, and whether an answer was found.

**Results:**

We analyzed 1305 patient-related questions sent to PubMed and/or UpToDate between October 1, 2005 and March 31, 2007 using our portal. A complete answer was found in 594/1125 (53%) questions sent to PubMed or UpToDate. A partial or full answer was obtained in 729/883 (83%) UpToDate searches and 152/242 (63%) PubMed searches (*P* < .001). UpToDate answered more questions than PubMed on all major medical topics, but a significant difference was detected only when the question was related to etiology (*P* < .001) or therapy (*P =* .002). Time to answer was 241 seconds (SD 24) for UpToDate and 291 seconds (SD 7) for PubMed.

**Conclusions:**

Specialists and residents in internal medicine generally use less than 5 minutes to answer patient-related questions in daily care. More questions are answered using UpToDate than PubMed on all major medical topics.

## Introduction

**Figure 1:**
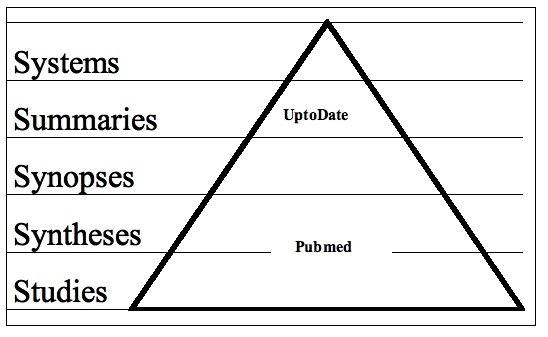
The “5S” levels of organization of evidence from health care research and the position of the studied information sources within the pyramid (after Haynes [1])

The use of Internet information sources for answering patient-related questions is taking an ever more important place in the daily practice of a physician. There are numerous sources available on the Internet. These sources can roughly be divided into five categories, as described by Haynes [1]. These five categories are arranged in a pyramid in the following top-down order, as depicted in Figure 1: systems (computerized, decision-support systems), summaries (evidence-based textbooks), synopses (evidence-based journal abstracts), syntheses (systematic reviews), and studies (original journal articles). 

UpToDate is an evidence-based, peer-reviewed information resource designed to provide information at the point of care [2]. PubMed is a search engine offering access to the Medline database [3].

From top to bottom, the information sources are less rigorously evaluated for evidence and take more time to evaluate for scientific rigor. On the other hand, it takes more time to establish the evidence. The sources at the top are therefore less up-to-date than sources at the bottom. Furthermore, the sources at the bottom are more abundant, being able to answer more questions. One should start searching preferably at the top, going from level to level when the source used did not provide the solution to the problem. From an evidence-based view, this is the best solution. As physicians usually spend less than 10 minutes to answer questions, this method would take too much time in the majority of cases [4-6]. When going down the pyramid of evidence takes too much time, it may be important to know at which level it is best to enter the pyramid. There may be certain topics (etiology, prognosis) that are difficult to find at a certain level and require a search that starts at a lower level. Furthermore, when certain topics are poorly addressed in information sources, this may give developers clues for enhancement of the information source. As there are links from our electronic patient record system to two major evidence-based information sources (PubMed and UpToDate), we conducted an observational study to determine how both sources are used in daily routine practice for answering patient-related questions. Our second target was the amount of time spent searching by hospital physicians.

## Methods

### Population and Measuring Tool

As part of an ongoing observation of medical information sources used to retrieve information, we developed a Web portal. This portal gives access to PubMed, UpToDate, Harrison’s Online, and a Dutch pharmacotherapy database. All residents and specialists in internal medicine selecting PubMed or UpToDate from our hospital information system were automatically linked to our portal.

### PubMed Interface

**Figure 2:**
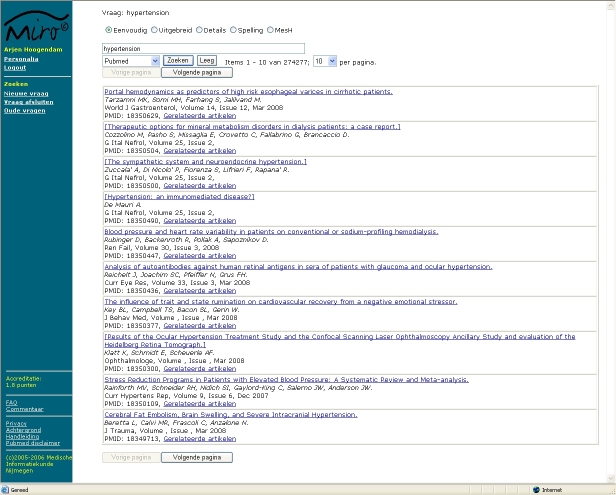
Screenshot of the portal: PubMed search result for “hypertension”

To enable the registration of all aspects regarding the use of PubMed, we built our own PubMed interface for accessing PubMed through e-utils [7]. E-utils gives access to full PubMed functionality. Query handling conducted by PubMed is identical to the original PubMed website, but e-utils delivers the data in XML to permit recording of the data in a database. The XML data need to be translated into Web pages to be readable for users. To mimic the functionality of PubMed, most of the special search options relevant for patient-related searches were copied in our interface: MeSH database, details, a selection of limits (publication date, publication type, human or animal, and age), and spelling. As shown in Figure 2, on the left of the page, the participant can choose to start searching for a new question, close the question, or re-open older questions (Nieuwe vraag, Vraag afsluiten, Oude vragen). There are links to background information (Achtergrond) and the manual (Handleiding). Search options are simple, advanced, details, check spelling, and MeSH database (Eenvoudig, Uitgebreid, Details, Spelling, and MeSH).

All queries were recorded as well as the use of the different search options, the articles that were selected for abstract reading, and the articles that were selected for full-text reading.

### Other Online Information Sources

**Figure 3:**
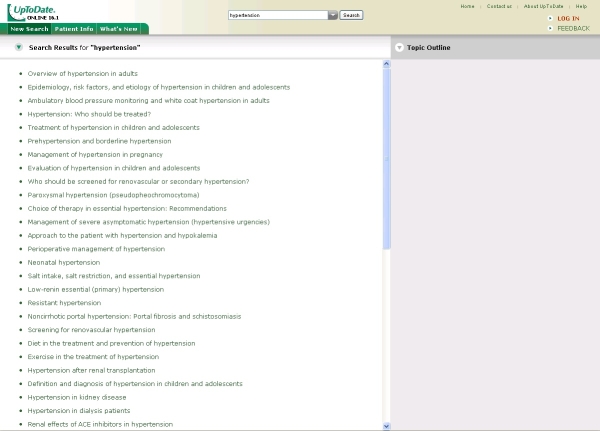
Screenshot of the UpToDate interface (Reproduced with permission from UpToDate, Rose BD, editor, UpToDate, Waltham, MA, 2008. Copyright 2008 UpToDate, Inc. [2])

As the other online sources do not permit direct access to their database, we linked directly to their website. The interface of UpToDate, therefore, was presented unaltered to the physician (Figure 3). After reading the information at the website, searchers returned to our own portal to answer questions regarding their search.

### Testing and Introduction

The portal was tested by direct observation using several user groups. After the testing phase, the program was introduced and tested by a select group of users during a period of 2 months. Starting October 2005, the portal was made publicly available. A manual is available from all screens in the portal. During the first year, all new users were asked if they needed help with the use of the portal. Participants received regular emails reminding them that help was available within the portal or that they could receive direct coaching.

### First Access

Upon accessing the database for the first time, the physician was asked to give informed consent to the observation of the search process. The physician was also presented with background information regarding our study and was urged to read the manual, which is available from every screen of the portal.

### Search Process

**Figure 4:**
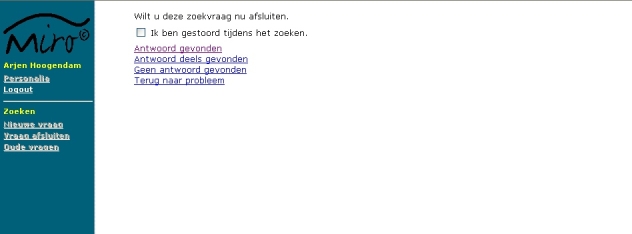
Screenshot of the page were participants could mark whether they were disturbed while searching, could select whether a complete, partial, or no answer was found, and could return to the problem

Every search was started by entering a query and selecting an information source. Search time was recorded by the monitoring program. Sending of the first query regarding a problem was marked as the start of the search. While searching, all queries were recorded by the portal. After completing the search, participants were asked whether they found no answer, a partial answer, or a full answer to their question; answering this question marked the end of the search (Figure 4).

**Figure 5:**
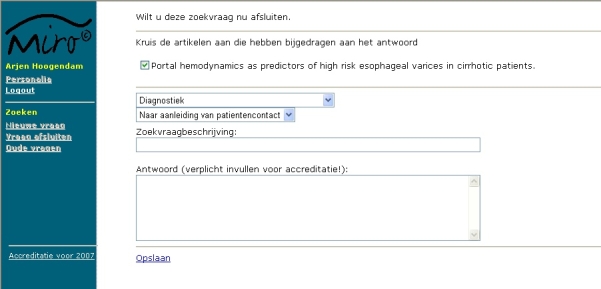
Screenshot of the page where details regarding the search could be provided

They were also asked to select the situation that led to the search (direct patient contact, patient rounds, scientific research, review/study, preparing talks, or not specified) and to place the topic into categories used by Hersh and Hickam and Haynes et al in previous studies (diagnosis, etiology, prognosis, therapy, side effects, complications, overview/review, mechanism, or unclear) [8,9]. Participants were given the option to provide additional data, including the question, the answer to the question, and whether articles selected for further reading contained information relevant to the question (Figure 5). The subject and the situation triggering the search could also be provided.

As multiple persons can access a single computer, sessions were automatically closed after 15 minutes of inactivity.

### Nonresponse

We intended to maximize the use of our computer portal. Physicians were encouraged to use the program as much as possible. At regular intervals, the database was checked to identify participants who infrequently provided details after searching. These participants were approached to determine the reason for nonresponse and were encouraged to improve their response. Nonresponse could be related to the participant but also to the monitoring system. We expected that physicians searching during daily medical care would not always be prepared to answer our questions directly after searching. Full-text articles and UpToDate were always opened in a separate pop-up window as most sites do not permit the opening of their Web pages within another frame. The Web page containing the questionnaire was available directly behind the pop-up windows. Forgetting to close the pop-up window after searching (and before closing the connection to the database) would lead to nonresponse. As both sources of nonresponse could lead to bias, we performed an additional check during the first year of our study. If participants did not fill in the questionnaire after searching, the questionnaire was repeated before the next search. As details regarding a former search are likely to become less reliable after some time, we intended to use the details provided within 24 hours after searching for a nonresponse bias analysis. After one year of monitoring, we had enough data to exclude nonresponse bias and removed the questionnaire before searching as it led to avoidance of the website.

### Selection of Queries

Only problems triggered by visit rounds or related to patient contact were included in our analysis. There were four different categories of searches: (1) searches that were completed with search-related details provided in one session, (2) searches with search-related details provided during a second session within 24 hours, (3) searches with search-related details provided during a second session after 24 hours, and (4) searches with no additional information provided. To minimize the risk of recall bias, only searches of the first category were included in our study. Searches of the second category were used for nonresponse bias analysis. The last two categories were excluded. The Dutch pharmaceutical database and Harrison’s Online cannot be considered as online evidence-based information sources because they do not link the text directly to literature references. Queries sent to these databases were therefore excluded from this study.

### Analysis

Whether an answer is partial or complete is a subjective qualification. We therefore combined partial and full answers when determining significance of our findings. Determining statistical significance was performed by the chi-square statistic. Statistical analysis was performed using SPSS 14.0 (SPSS Inc, Chicago, IL, USA).

## Results

**Figure 6:**
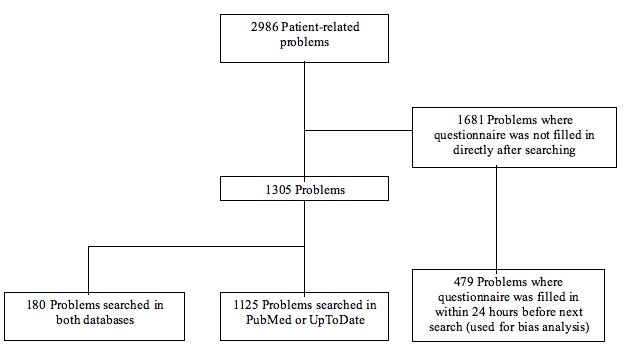
Selection of problems for analysis

Participants used our portal for 2986 patient-related questions. These questions were sent by 40 residents and 30 specialists in internal medicine from October 1, 2005 to March 31, 2007. There were 1305 searches selected for analysis, according to the inclusion criteria (Figure 6).

Aspects of searches conducted in a single database are shown in Table 1. UpToDate was the most popular database with 883/1125 (78%) questions. The most popular topics were diagnosis, etiology, and therapy, with 924/1125 (82%) questions. Full answers were provided to 594/1125 (53%) questions. A partial or full answer was obtained in 729/883 (83%) UpToDate searches and 152/242 (63%) PubMed searches (*P* < .001).

Analysis of searches answered during a second session within 24 hours found partial or full answers obtained by 260/300 (87%) UpToDate searches and 115/179 (64%) PubMed searches, showing that there was no negative response bias.

The average time spent searching online medical sources was 252 seconds. Time to answer was 291 seconds (SD 24) for searches conducted in PubMed and 241 seconds (SD 7) for searches conducted in UpToDate.

Data concerning questions sent to both databases compared with questions sent to a single database are shown in Table 2. Consultation of UpToDate occurred frequently after searching in PubMed, in 119/361 (33%) searches, and resulted in more partial and full answers than the consultation of PubMed alone. Searching PubMed after consulting UpToDate occurred in 61/944 (6%) searches, but did not result in more partial or full answers than the consultation of UpToDate alone.

The relationship between search topic and answers found is shown in Table 3. Queries sent to UpToDate resulted in a higher percentage of answers compared with PubMed, regardless of the subject. This difference was significant in queries concerning etiology and therapy.

The use of information sources by residents and specialists is shown in Table 4. Residents used UpToDate for 579/669 (87%) questions, in contrast to specialists, who used UpToDate for 304/456 (67%) questions. PubMed searches were equally successful for both specialists, but UpToDate provided relatively more answers to residents.

**Table 1:** Aspects of questions that were sent to only one of the two databases (N = 1125)

	PubMed (N = 242)	UpToDate (N = 883)	*χ*^2^^†^	*P*
	No. (%)^*^	No. (%)^*^		
**Answer**			54	< .001
No answer found	90 (37)	154 (17)		
Partially answered	68 (28)	219 (25)		
Fully answered	84 (35)	510 (58)		
**Subject**				
Diagnosis	51 (21)	400 (45)	46.41	< .001
Etiology	70 (29)	219 (25)	1.69	.19
Prognosis	3 (1)	8 (1)	0.01	.92
Therapy	41 (17)	143 (16)	0.08	.78
Side effects	14 (6)	12 (1)	16.48	< .001
Complications	17 (7)	33 (4)	4.83	.03
Overview/Review	40 (17)	61 (7)	21.51	< .001
Mechanism	5 (2)	3 (0.3)	5.76	.02
Unclear	1 (0.4)	4 (0.4)	0.21	.64

^*^Percentages may not add to 100% due to rounding.

^†^Chi-square of difference between UpToDate and PubMed.

**Table 2:** Comparison of answers to questions sent to a single database and to both databases (N = 1305)

Primary Information Source	Secondary Information Source	Answer^*^		
		None Found	Partially Answered	Fully Answered
		n/N (%)	n/N (%)	n/N (%)
PubMed	None	90/242 (37)	68/242 (28)	84/242 (35)
PubMed	UpToDate	20/119 (17)	47/119 (40)	52/119 (44)
UpToDate	None	154/883 (17)	219/883 (25)	510/883 (58)
UpToDate	PubMed	20/61 (33)	26/61 (43)	15/61 (25)

^*^Percentages may not add to 100% due to rounding.

**Table 3:** Number and percentage of partial or full answers found to questions sent to only one of the two databases, by subject (N = 1125)

Subject	PubMed	UpToDate	*χ*^2^_1_^*^	*P*
	n/N (%)	n/N (%)		
Diagnosis	38/51 (75)	339/400 (85)	3.46	.06
Etiology	38/70 (54)	175/219 (80)	17.97	< .001
Prognosis	2/3 (67)	7/8 (88)	0.01	.94
Therapy	24/41 (59)	117/143 (82)	9.64	.002
Complications and side effects	22/31 (71)	37/45 (82)	1.34	.25
Other^†^	28/46 (61)	54/68 (79)	4.67	.03

^*^Chi-square of difference between PubMed and UpToDate in partial and full answers found.

^†^Mechanism, unclear, and overview/review combined.

**Table 4:** Number and percentage of partial or full answers found by specialists and residents to questions sent to only one of the two databases

	Resident	Specialist	*χ*^2^_1_^*^	*P*
	n/N (%)	n/N (%)		
PubMed	57/90 (63)	95/152 (63)	0.02	.90
UpToDate	488/579 (84)	241/304 (79)	3.47	.06

^*^Chi-square of difference between residents and specialists in partial and full answers found in PubMed and UpToDate.

## Discussion

This is an observational study that delivers valuable data regarding the actual use of PubMed and UpToDate during daily medical practice. Our study shows that participants were able to find full answers to 53% of their questions using our portal, which is comparable to results found in other studies [5,10].

Physicians spend less than 5 minutes on average searching for online information. Previous studies have pointed out that the use of evidence at the point of care is closely related to the time needed to answer the question. Most of the questions generated by physicians can be answered, but it is time consuming and expensive to do so [11,12]. The time used for searching online information sources was shorter than that found in other studies [5,6,13,14] in which conditions did not always reflect daily care, but comparable to the study by van Duppen et al performed during daily patient visits [15].

Participants preferentially used UpToDate and succeeded in answering more patient-related questions during daily medical care using UpToDate than using PubMed. This is comparable to previous research in which UpToDate is the preferred information source over PubMed and is perceived as equally or more useful for answering patient-related questions [16-19].

Schilling et al suggested that PubMed and UpToDate are used by residents as complementary sources [17]. UpToDate would be more suitable for general questions about well established evidence, and PubMed would be more suitable for specific questions. However, physicians interviewed by Ely et al stated that common conditions are not searched because the answers are already known [18]. But, it is just as likely that common conditions trigger complex questions and rare conditions trigger general questions. We did not rate the complexity of the questions or motivations for selecting a particular database, but clinical experience and conducting searches in both databases are likely to be related to question complexity. When both databases were used, the consultation of UpToDate after PubMed occurred more frequently and resulted in more partial or full answers in comparison to consultation of UpToDate followed by PubMed and PubMed alone. This would not be the case if PubMed was used primarily for complex questions with answers that were not likely to be found in UpToDate. Our findings show that starting the search with UpToDate, followed by consultation of PubMed if the answer is not satisfactory, is a sensible strategy. This is consistent with the advice given by Haynes [1]. If the complexity of questions plays a crucial role in the choice of an information source, the choice is influenced by experience. As it is likely that specialists have more detailed knowledge than residents, we used professional status as an indicator of question complexity. Our data show that there was no difference in PubMed search results between residents and specialists. Residents were able to answer more questions using UpToDate; however, this difference is not significant and too small to be of concern in daily practice. PubMed was used relatively more frequently by specialists than by residents. Professional status is likely to play a role in the choice of an information source, but it is not reflected in a substantial difference in search results. Professional status, therefore, is no argument for choosing a particular information source.

Our data show that questions sent to UpToDate retrieved more answers than questions sent to PubMed regardless of major medical topic. This difference was only significant in etiology and therapy, but sample size is insufficient to detect significance in other medical topics. Based on our data, there is no reason to start searching on a lower level of the evidence-based pyramid for any major medical topic, but it is sensible to use UpToDate as the primary information source.

Ely et al identified 59 obstacles when searching for evidence-based answers to doctors’ questions [20]. Among the most salient were failure of the resource to address the topic, inadequate time to search for information, and inadequate synthesis of multiple bits of evidence into a clinically useful statement. Online textbooks provide information that is synthesized and displayed in a text that can be scanned within a couple of minutes, but failure to address the topic is the limiting factor. Search time and scattering of evidence over multiple articles are the limiting factors for PubMed. This, combined with the fact that physicians spend less than 5 minutes to find an answer during daily medical care, makes PubMed an unsuitable information source to use. Conducting a thorough search takes nearly 30 minutes [21]. This is the most likely explanation why UpToDate is the primary information source and performs better at the point of care in our study and other studies [16-19]. Improvements in PubMed must therefore be aimed at trying to create search methods that are targeted to a maximum search time of 5 minutes, including time needed for evaluation of the literature. Improvements in search methods that are aimed at significantly reducing search time are likely to increase the effectiveness of PubMed for patient-related questions during daily medical care.

### Limitations

This study was performed in a single hospital where specialists and residents are accustomed to accessing PubMed and UpToDate as primary information sources. There are many more evidence-based information sources available on the Internet. For our observation, we chose to use the information sources that our population was familiar with, limiting the generalizability of our results.

Optimal testing of the performance of medical information sources requires taking the physician out of daily practice as physicians will not be prepared to look up answers in several databases and answer additional questionnaires during working hours. Most studies, therefore, resort to observation in laboratory situations or questionnaires without direct observation [22]. As PubMed is likely to answer most of the questions if the search time is unlimited, testing PubMed out of daily practice without time constraint is meaningless for daily care use. We used a novel approach that combined observation with post-search questionnaires. We consider PubMed and UpToDate as reliable information sources, but there is limited information that compares their usefulness in daily use. Physicians working at our hospital are very familiar with these sources; PubMed and UpToDate are therefore ideal for an observational study regarding their everyday use. There are several limitations to an observational study that apply to our study as well. We could not influence the information source approached or check whether the answer would be found in a second database in all questions. This makes a direct comparison of the information sources impossible.

We rebuilt most of the functionality of PubMed in our interface. However, exact mimicry of the website was not allowed by legal and ethical issues. Users could provide comments to the portal but did not report that the use of our interface was more difficult than the original PubMed interface.

The fact that physicians report that they have found an answer is not a guarantee that the answer really has been found. Physicians tend to overestimate the quality of the information retrieved through searching. Previous studies have shown that correct answers before searching can be incorrectly altered by searching online information sources [14,23]. Whether a partial or full answer is found is a subjective interpretation. The qualification should, however, reflect satisfaction of the participant with the obtained answer.

In many questions, the questionnaire was not filled in after searching. The major reason is opening of multiple Web pages on the screen, causing the monitoring program to disappear in the background. This, in turn, resulted in participants forgetting to answer the required information after the search within the time limit of 15 minutes. We also suspected that physicians would be reluctant to spend additional time answering search-related questions during daily care. It is likely that more complex questions leading to no answer after extensive searching will result in nonresponse. To detect whether this noncompliance would lead to a nonresponse bias, we performed a secondary analysis regarding queries answered during a second session within 24 hours. The results were comparable, showing that question complexity itself was not a reason for nonresponse.

PubMed is our default database for searching, so the use of PubMed might be overestimated. We asked whether participants were interrupted while searching, but we did not exclude these searches because we consider disturbances part of one’s daily routine. As we did not ask what database gave the answer to the question, it is impossible to identify which database contributed most to the answer when multiple sources were used. For this study, we assumed that the intention for consulting a second database was to improve the answer found in the first information source.

### Conclusions

Our study makes a contribution in observing hospital physicians in their daily routine solving patient-related questions. We have shown that answers to questions posed during daily medical care are more likely to be answered by UpToDate than PubMed, regardless of the topic of the search. Physicians trying to answer patient-related questions use less than 5 minutes to search for an answer during daily medical care. Improving medical information sources should be aimed at delivering an answer within 5 minutes as this is the average time a hospital specialist spends finding an answer at the point of care. Future research should be aimed at comparing more information sources at different levels of the evidence pyramid. Question complexity may play a role in the choice of where to enter the hierarchy of evidence-based sources. Analysis of query content and the search process should reveal more information to improve PubMed as a search tool for daily medical care.
